# Differential Analysis of Longitudinal Methicillin-Resistant Staphylococcus aureus Colonization in Relation to Microbial Shifts in the Nasal Microbiome of Neonatal Piglets

**DOI:** 10.1128/mSystems.00152-21

**Published:** 2021-07-20

**Authors:** Shriram Patel, Abel A. Vlasblom, Koen M. Verstappen, Aldert L. Zomer, Ad C. Fluit, Malbert R. C. Rogers, Jaap A. Wagenaar, Marcus J. Claesson, Birgitta Duim

**Affiliations:** a APC Microbiome Ireland, University College Cork, Cork, Ireland; b Department of Infectious Diseases and Immunology, Faculty of Veterinary Medicine, Utrecht University, Utrecht, the Netherlands; c Department of Medical Microbiology, University Medical Centre Utrecht, Utrecht, the Netherlands; d Wageningen Bioveterinary Research, Lelystad, the Netherlands; Oregon State University

**Keywords:** MRSA, *Staphylococcus aureus*, colonization, microbial shifts, porcine nasal microbiome

## Abstract

Methicillin-resistant Staphylococcus aureus (MRSA) is an important human pathogen and often colonizes pigs. To lower the risk of MRSA transmission to humans, a reduction of MRSA prevalence and/or load in pig farms is needed. The nasal microbiome contains commensal species that may protect against MRSA colonization and may be used to develop competitive exclusion strategies. To obtain a comprehensive understanding of the species that compete with MRSA in the developing porcine nasal microbiome, and the moment of MRSA colonization, we analyzed nasal swabs from piglets in two litters. The swabs were taken longitudinally, starting directly after birth until 6 weeks. Both 16S rRNA and *tuf* gene sequencing data with different phylogenetic resolutions and complementary culture-based and quantitative real-time PCR (qPCR)-based MRSA quantification data were collected. We employed a compositionally aware bioinformatics approach (CoDaSeq + rmcorr) for analysis of longitudinal measurements of the nasal microbiota. The richness and diversity in the developing nasal microbiota increased over time, albeit with a reduction of *Firmicutes* and *Actinobacteria*, and an increase of *Proteobacteria*. Coabundant groups (CAGs) of species showing strong positive and negative correlation with colonization of MRSA and S. aureus were identified. Combining 16S rRNA and *tuf* gene sequencing provided greater Staphylococcus species resolution, which is necessary to inform strategies with potential protective effects against MRSA colonization in pigs.

**IMPORTANCE** The large reservoir of methicillin-resistant Staphylococcus aureus (MRSA) in pig farms imposes a significant zoonotic risk. An effective strategy to reduce MRSA colonization in pig farms is competitive exclusion whereby MRSA colonization can be reduced by the action of competing bacterial species. We complemented 16S rRNA gene sequencing with Staphylococcus-specific *tuf* gene sequencing to identify species anticorrelating with MRSA colonization. This approach allowed us to elucidate microbiome dynamics and identify species that are negatively and positively associated with MRSA, potentially suggesting a route for its competitive exclusion.

## INTRODUCTION

Staphylococcus aureus is an opportunistic pathogen that can colonize and infect humans and animals. The anterior nares are among the host sites that S. aureus can colonize. Methicillin-resistant strains of S. aureus (MRSA) have been described since 1961 (1) and frequently carry additional resistance determinants (2). Since the discovery of livestock associated methicillin-resistant S. aureus (LA-MRSA) in pig farms (3, 4), such strains have been reported all over the world (5). In 2015, 99.5% of the tested slaughter pigs in the Netherlands were LA-MRSA positive (6). Between 2009 and 2018, a high LA-MRSA prevalence was observed in fattening pigs in some European Union member states (7). This reservoir of antimicrobial-resistant staphylococcal strains in farm animals creates a risk of zoonotic transfer.

Fortunately, research has shown LA-MRSA mostly transfers from pigs to humans and less frequently from human to human (8). Currently, it is estimated that 15% of the MRSA skin and soft tissue infections in the community, compared to 1 to 2% of the hospital-acquired cases, are LA-MRSA associated (2). Contamination of humans with LA-MRSA occurs predominantly through occupational exposure (8). However, recent reports point at the risk of human-adapted LA-MRSA sublineages (9). Therefore, reducing the number of LA-MRSA-positive pig herds or reducing the load of LA-MRSA in pigs could reduce LA-MRSA transfer to susceptible people in the population.

Attempts to reduce LA-MRSA in pig farms are estimated to be very costly (10), and their effectivity over time has not been studied. Although the reduction of antimicrobial usage resulted in a decrease of resistance levels in E. coli (11), the prevalence of LA-MRSA in pig farms remained stable (6, 12), indicating that other strategies are needed to reduce the colonization of LA-MRSA in pigs.

One strategy, competitive exclusion, consists of introducing microorganisms that will effectively out-compete a species in the host microbiome. Competitive exclusion has been famously applied to control Salmonella in poultry (13, 14). Attempts have been made to alter the human nasal microbiome to make it hostile to MRSA. For example, several studies have shown that Staphylococcus epidermidis can destroy MRSA biofilms and protected against nasal colonization with S. aureus (15, 16). In the 1960s, less pathogenic S. aureus strains were used to prevent colonization of more harmful S. aureus strains in the noses of infants in nurseries (17, 18). In other body sites probiotic strategies against MRSA are more frequently studied, for example, the usage of bacillus probiotics to reduce the S. aureus load in the intestine of humans (19).

Little, however, is known about similar MRSA reduction strategies in pigs. Espinosa-Gongora et al. described 20 operational taxonomic units (OTUs) from nasal samples from pigs 3 weeks before slaughter that might be negatively associated with carrying S. aureus, based on 16S rRNA gene sequencing and mapping against the Ribosomal Database Project (RDP) (20). Others authors have described a lack of difference between MRSA carriers and noncarriers (21).

The aim was to identify bacteria that are antagonistic to MRSA colonization in pigs. Therefore, we studied the dynamics of the nasal microbiome of neonatal pigs in relation to S. aureus carriage, using two marker genes, the 16S rRNA gene and the elongation factor thermo unstable (EF-TU) encoding gene, *tuf*. *tuf* sequencing was included, as it provides improved resolution of Staphylococcus species (22, 23). The sequencing data were complemented with culturing and quantitative PCR data to provide additional resolution to the S. aureus and MRSA abundance at each time point. We determined the species residing in the porcine nasal microbiome and the longitudinal dynamics of their microbial taxa, while identifying multiple species anticorrelating to MRSA and S. aureus.

## RESULTS

### Cohort characteristics and sequencing summary.

A total of 104 samples from 8 piglets across 13 different time points were collected to study the association of the microbiota with carriage of MRSA and S. aureus in the nasal cavity of growing piglets. Of these, 39 samples were detected as S. aureus-positive based on quantitative real-time PCR (qPCR) and cultural enumeration data, but none of the piglets were found to be S. aureus-negative on all sampling occasions (Fig. S1). Most of the piglets became positive after day 4, with the exception of piglets from litter A, which were intermittently positive during initial time points. Piglets from litter A were more often positive for S. aureus than piglets from litter B (24/52 versus 15/52 samples). The mean number of S. aureus in positive samples was 4.60 × 10^4^ and 3.0 × 10^4^ log CFU-equivalents (CFUeq)/swab for litter A and litter B, respectively. Notably, 31 of the 39 S. aureus-positive nasal samples were also MRSA-positive. MRSA was detected in 20 S. aureus-negative samples (Fig. S1). The mean number of MRSA CFU in positive samples was 138 and 19 CFU/swab in piglets from litter A and litter B, respectively.

10.1128/mSystems.00152-21.1 FIG S1 Summary of the number of CFUeq of S. aureus and CFU of MRSA in nasal swabs of the individual piglets. Download FIG S1, PDF file, 0.3 MB. Copyright © 2021 Patel et al. 2021 Patel et al. https://creativecommons.org/licenses/by/4.0/ This content is distributed under the terms of the Creative Commons Attribution 4.0 International license.

Microbiome analysis was carried out on 7.07 and 7.34 million error-corrected, nonchimeric amplicon sequence variant (ASV) reads with a mean count of 80,282 ± 26,624 standard deviation (SD) and 71,272 ± 28,735 SD reads for the 16S and *tuf* data sets, respectively. In addition, 4 negative-control samples were sequenced, but considerably fewer error-corrected reads were generated, with an average of 401 and 2,351 reads for the 16S and *tuf* data sets, respectively (Figure S2a). Overall, 2,787 unique 16S ASVs were identified, 23 of which were detected as potential contaminants. For *tuf*, 39 of the 1,278 ASVs were identified as potential contaminants and were removed. However, this did not have any effect on overall sequencing depth (Figure S2b). Samples with less than 5,000 (*n* = 8) reads in the 16S and 13,000 (*n* = 1) reads in the *tuf* data sets were excluded from further analysis. The numbers of reads retained after abundance-based filtering, i.e., after excluding ASVs present in less than 10% of samples with less than 0.001% abundance, were 95.24 ± 8.75% and 97.17 ± 6.45% for 16S and *tuf* sequencing, respectively (Figure S2b). Thus, the contribution of the excluded ASVs to the overall number of reads per sample was found to be very small or negligible.

10.1128/mSystems.00152-21.2 FIG S2 (A) Summary of the number of sequences assigned to samples and mix controls for the 16S and *tuf* data sets. (B) Summary of the sequencing depth before and after contaminant removal and after ASV filtering (i.e., prevalence- and abundance-based filtering). Download FIG S2, PDF file, 0.5 MB. Copyright © 2021 Patel et al. 2021 Patel et al. https://creativecommons.org/licenses/by/4.0/ This content is distributed under the terms of the Creative Commons Attribution 4.0 International license.

### General population structure of piglet nasal microbiota.

At phylum level in the 16S data set, a total of 25 unique phyla, including *Proteobacteria*, *Firmicutes*, *Actinobacteria*, *Bacteroidetes*, and *Euryarchaeota* were observed, with *Proteobacteria* (39.5%) being the most abundant, followed by *Firmicutes* (30.1%) and *Actinobacteria* (25.2%), across each time point (Fig. S3A), accounting for 94.8% of all reads. The most abundant taxa at the genus level were *Moraxella* (29.7%), *Rothia* (23.9%), Streptococcus (11.5%), and *Mannheimia* (6.8%), while *Clostridium*, *Aerococcus*, *Bergeyella*, *Corynebacterium*, Staphylococcus, *Lactobacillus*, and *Porphyromonas*, each accounting for >1% of total bacterial abundance (Fig. S3C and Fig. S4A).

10.1128/mSystems.00152-21.3 FIG S3 Distribution of the top most abundant (A) 16S phylum (B) *tuf* phylum, (C) 16S genus, and (D) *tuf* genus across the timepoints. Download FIG S3, PDF file, 0.9 MB. Copyright © 2021 Patel et al. 2021 Patel et al. https://creativecommons.org/licenses/by/4.0/ This content is distributed under the terms of the Creative Commons Attribution 4.0 International license.

10.1128/mSystems.00152-21.4 FIG S4 Longitudinal changes in relative abundance of the top most abundant genus in the (A) 16S and (B) *tuf* data sets. Download FIG S4, PDF file, 0.6 MB. Copyright © 2021 Patel et al. 2021 Patel et al. https://creativecommons.org/licenses/by/4.0/ This content is distributed under the terms of the Creative Commons Attribution 4.0 International license.

In contrast, *tuf* sequences were classified to only four phyla, with *Firmicutes* (86.3%) and *Proteobacteria* (13.6%) being the most abundant (Fig. S3B), whereas Streptococcus (46.2%), Staphylococcus (17.1%), *Moraxella* (13.6%), *Enterococcus* (9.0%), *Micrococcus* (2.1%) and *Gemella* (1.5%) were the most dominant genera (Fig. S3D and S4B). The relative abundances of the top most-abundant phyla and genera from both 16S and *tuf* data sets are reported in Fig. S3 and S4.

### *tuf* gene sequencing provided species-level resolution of Staphylococcus taxa.

**FIG 1:**
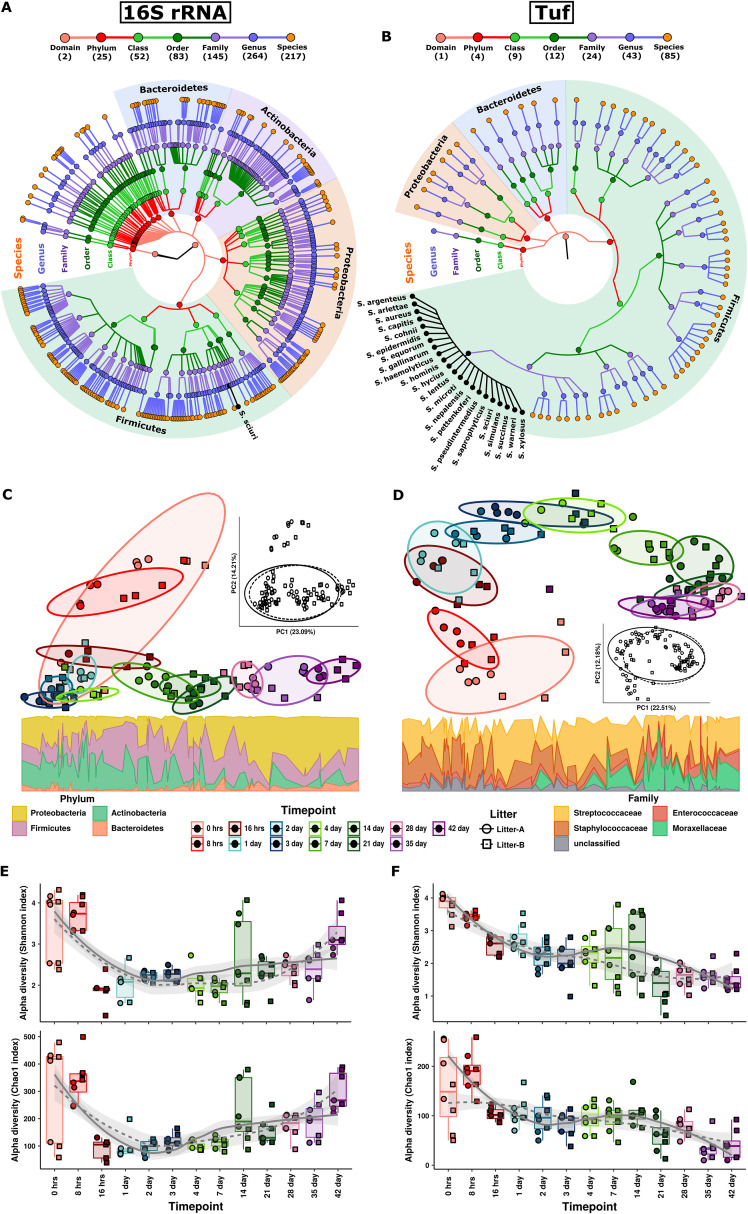
Longitudinal changes in the piglet nasal microbiome structure and community diversity. (A and B) Taxonomic tree structure of the microbial community as revealed by (A) 16S rRNA gene and (B) *tuf* gene sequencing. From the inner to outer circle, the taxonomic levels range from domain to specie levels of taxa. Different colors of dots indicate different taxonomy levels according to the color key shown. Numbers in parentheses indicate the total number of unique taxonomies detected at each level. Different colors in the background represents phylum-level taxa. Dots, lines, and name of the species in black represent species identified from Staphylococcus taxa. (C and D) PCA analysis based on an Aitchison distance matrix shows distinct clustering of the samples based on time points from birth to day 42 with less but significant litter effect on overall microbiome composition in (C) 16S rRNA and (D) *tuf* gene sequencing data. The inset PCoAs are labeled by litter membership. The bottom panel shows variation of phylum- and family-level microbiome composition along the PC1 axis in 16S rRNA and *tuf* gene sequencing, respectively. (E and F) Box plots show the Shannon and Chao1 alpha diversity measures according to (E) 16S rRNA and (F) *tuf* gene alpha diversity. Nonlinear trends in alpha diversity from birth to day 42 were identified by fitting loess regression splines from the ggplot2 package.

It has previously been noted that the V4 hypervariable region of the 16S rRNA gene alone is not sufficiently discriminative for the identification of species within the Staphylococcus genus (24, 25). Therefore, to increase the resolution of the Staphylococcus genus, which was one of the aims of the current study, we also carried out amplicon sequencing of the *tuf* gene, which better discriminates between different species of Staphylococcus, Streptococcus, and *Enterococcus* (26, 27). In particular, *tuf* gene sequencing led to the identification of 22 different Staphylococcus species, while 16S rRNA gene sequencing only detected the Staphylococcus sciuri species (Fig. 1A and B). When examining the data at the even more granular ASV level, we identified 137 and 10 different sequence variants belonging to Staphylococcus taxa from the *tuf* and 16S data sets, respectively (Fig. S5). As expected, the abundance of ASVs assigned to the Staphylococcus genus in samples sequenced for both 16S rRNA and the *tuf* gene correlated significantly (rmcorr coefficient [r_rm_] = 0.75, confidence interval [CI] = 0.68 to 0.86, *P* = 1 × e^−17^).

10.1128/mSystems.00152-21.5 FIG S5 Relative abundance of Staphylococcus ASVs as identified from the 16S and *tuf* data sets. Download FIG S5, PDF file, 0.6 MB. Copyright © 2021 Patel et al. 2021 Patel et al. https://creativecommons.org/licenses/by/4.0/ This content is distributed under the terms of the Creative Commons Attribution 4.0 International license.

### Longitudinal development of piglet nasal microbiota.

The composition of the piglet nasal microbiota based on 16S rRNA gene sequencing shows clear segregation of the samples based on time points (Fig. 1C). The piglets exhibited a gradual shift in collective microbiome composition from day 0 to day 42, with permutational multivariate analysis of variance (PERMANOVA) analysis showing a significant time point-associated variance on microbiota structure (R^2^ = 0.55, *P* = 1 × e^−4^). In alignment with the 16S rRNA gene data, significant changes in community membership and a clear structural shift from day 0 to day 42 (PERMANOVA: R^2^ = 0.49, *P* = 1 × e^−4^) were also apparent from *tuf* analysis (Fig. 1D). Litter membership explained significant but less of the variation of the microbiome community structure (PERMANOVA: 16S R^2^ = 0.03, *P* = 1 × e^−4^; tuf R^2^ = 0.04, *P* = 1 × e^−4^). High interindividual differences and separate clustering observed for 0-h and 8-h samples might be related to the effect of unstable microbiota derived from fecal or soil contamination in newborn piglets (Fig. S4A and S6). Interestingly, we observed that as the piglets age, *Proteobacteria* (i.e., *Moraxellaceae* in *tuf*) increase, while taxa belonging to *Firmicutes* (i.e., *Staphylococcaceae* in *tuf*) decrease (Fig. 1C and D).

10.1128/mSystems.00152-21.6 FIG S6 Presence-absence-based heatmap of the top 368 ASVs in the 16S data. The highlighted red block indicates ASVs that are unique to the first two time points and belong to the genera *Clostridia, Lactobacillus*, and *Aerococcus*. Download FIG S6, PDF file, 0.8 MB. Copyright © 2021 Patel et al. 2021 Patel et al. https://creativecommons.org/licenses/by/4.0/ This content is distributed under the terms of the Creative Commons Attribution 4.0 International license.

We observed higher microbiota alpha diversity in 0-h and 8-h samples in both 16S and *tuf* data sets, but these levels then dropped dramatically at 16 h, after which the diversity gradually increased for 16S data (Fig. 1E), while it successively decreased for *tuf* data after a peak at day 7 (Fig. 1F). There was a high presence of the genus *Clostridium*, a strict anaerobe often described in the gut, at 0 h and 8 h (Fig. S6). The higher microbial richness and diversity observed in the 0-h and 8-h samples might be related to bacteria introduced from the birth canal or fecal or soil contaminants in the newborn piglets. These observations were used as evidence to exclude the first two time points from the anticorrelative analysis against S. aureus. Next, we investigated dynamic changes in alpha diversity over time, and we found a significant increase in chao1 (richness; 16S: r_rm_ = 0.67, CI = 0.50 to 0.80; tuf: r_rm_ = −0.62, CI = −0.73 to −0.45) and Shannon index (evenness; 16S: r_rm_ = 0.50, CI = 0.28 to 0.66; tuf: r_rm_ = −0.51, CI = −0.62 to −0.38) with time after exclusion of 0-h and 8-h samples. The negative alpha diversity trend observed in the *tuf* data set may be explained by the reduced abundance of the taxa-rich *Firmicutes* phylum in nasal microbiota of growing piglets at later time points (Fig. 1F and Fig. S3B).

**FIG 2:**
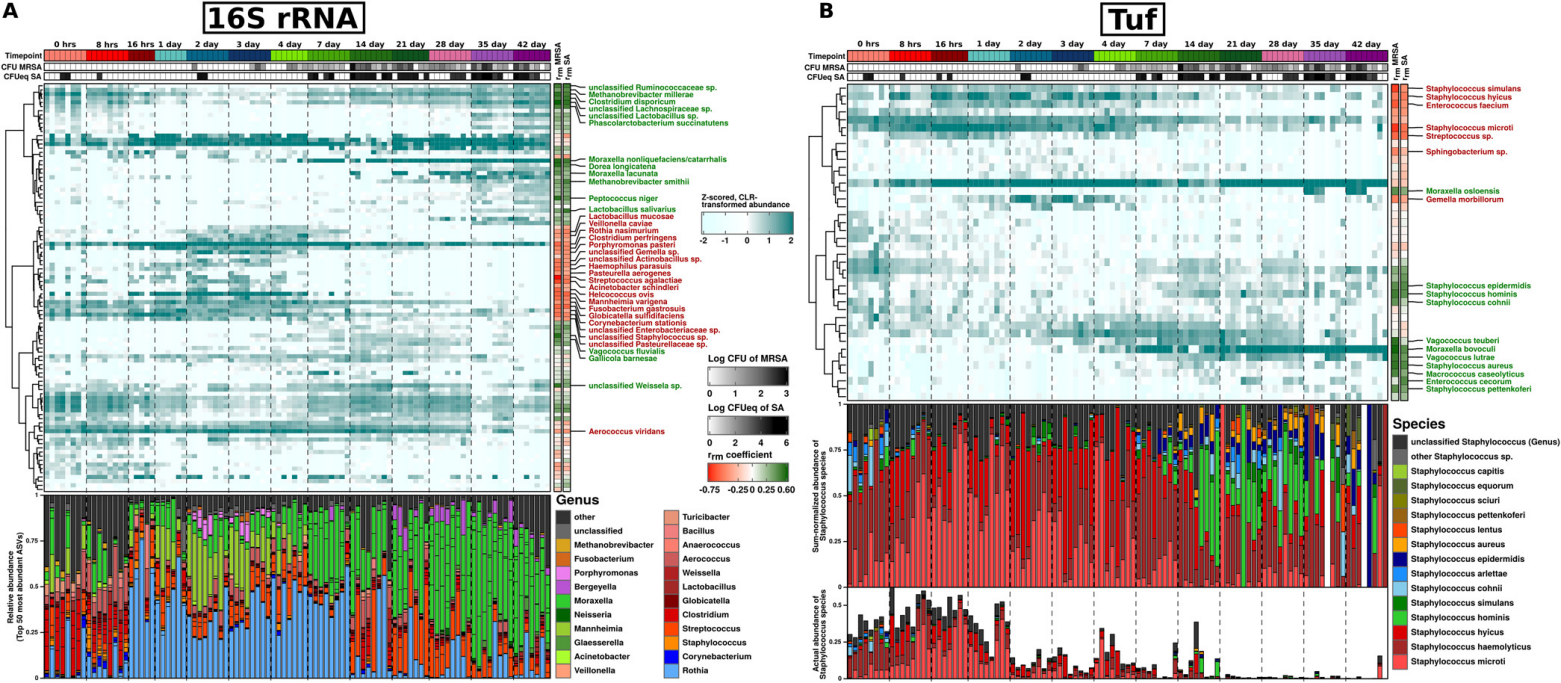
Community-level changes in microbial taxa associated with nasal colonization of MRSA and S. aureus over time. (A and B) The heatmap shows the association of CFUeq of S. aureus/CFU of MRSA with species summarized microbial taxa in (A) 16S rRNA and (B) *tuf* gene sequencing data and culture results. Columns (samples) are ordered by time points, and rows (species) are ordered by a Spearman correlation distance matrix and ward linkage hierarchical clustering. Time points and density of CFUeq of S. aureus/CFU of MSRA are depicted as the top annotation. The strength of correlation of taxa with MRSA/S. aureus nasal colonization as measured by the rmcorr package is displayed as sidebars (rrm coefficient). Taxa showing significant correlation (adj. *P* value < 0.05) with MRSA/S. aureus colonization are labeled as text annotations in green (positive correlation) and red (negative correlation). The overall relative abundance of the top 50 most abundant ASVs colored based on their genus is noted in the bottom annotation in 16S rRNA data. The actual relative abundance of Staphylococcus taxa (bottom) and sum-normalized relative the abundance of Staphylococcus taxa are noted in the bottom annotation in the *tuf* gene sequencing data.

The age-based dynamic changes of the microbiome compositions were further evaluated at a lower taxonomic level. Using 16S data, we found there were 22 genera markedly changed among the top 50 abundant ASVs. The relative abundance of ASVs from the genus *Rothia* (and Rothia nasimurium at species level) increased from 16 h to 7 days but subsequently decreased after 14 days. Decreases in abundance of *Rothia* was accompanied by increases of *Moraxella* and Streptococcus genera (Fig. 2A). In the *tuf* data, Staphylococcus accounted for more than 25% of the bacterial sequences until the age of 1 day but decreased dramatically from day 2 to day 14, which agrees with the 16S data. Of the 22 identified Staphylococcus species, S. microti (6.4%), S. haemolyticus (3.2%) and S. hyicus (3.2%) were the most abundant, while S. hominis, S. simulans, S. cohnii, S. arlettae, S. epidermidis, and S. aureus each accounted for approximately 0.1% of total bacterial abundance (Fig. 2B, bottom annotation;).

### Association of microbiota with MRSA and S. aureus carriage.

We subsequently investigated whether the abundance of nasal microbiota can predict MRSA or S. aureus nasal colonization, using repeated measure correlation analysis. Here, we identified 28 genera that were significantly associated with colonization of MRSA, of which *Sphingobacterium*, Pseudomonas, *Rothia*, Staphylococcus, *Gemella*, and *Alloiococcus* were strongly negatively correlated with MRSA colonization (r_rm_ > −0.5; all adjusted [adj.] *P* values < 0.05), while *Oscillospira*, *Dorea*, *Peptococcus*, *Lactobacillus*, *Coprococcus*, and *Methanobrevibacter* were strongly positively correlated (r_rm_ > 0.5; adj. *P* value < 0.05) (Table S1). In terms of S. aureus colonization, of the total 21 significantly associated genera, Staphylococcus (r_rm_ = −0.49) and *Actinobacillus* (r_rm_ = −0.48) were the most negatively correlated, and *Oscillospira*, *Eubacterium*, *Blautia*, and *Methanobrevibacter* were the most positively correlated (r_rm_ > 0.5; adj. *P* value < 0.05) taxa (Table S1). Comparable results were obtained when analyzing genus-level *tuf* data, with Staphylococcus, *Gemella*, and *Sphingobacterium* (r_rm_ > −0.45; adj. *P* value < 0.05) negatively correlated and *Moraxella* (r_rm_ = 0.61) and *Vagococcus* (r_rm_ = 0.58) positively correlated with MRSA colonization (Table S1).

10.1128/mSystems.00152-21.9 TABLE S1 Genus- and species-level taxa significantly associated with MRSA/S. aureus colonization using rmcorr analysis in the 16S and *tuf* data sets. Download Table S1, XLSX file, 0.06 MB. Copyright © 2021 Patel et al. 2021 Patel et al. https://creativecommons.org/licenses/by/4.0/ This content is distributed under the terms of the Creative Commons Attribution 4.0 International license.

**FIG 3:**
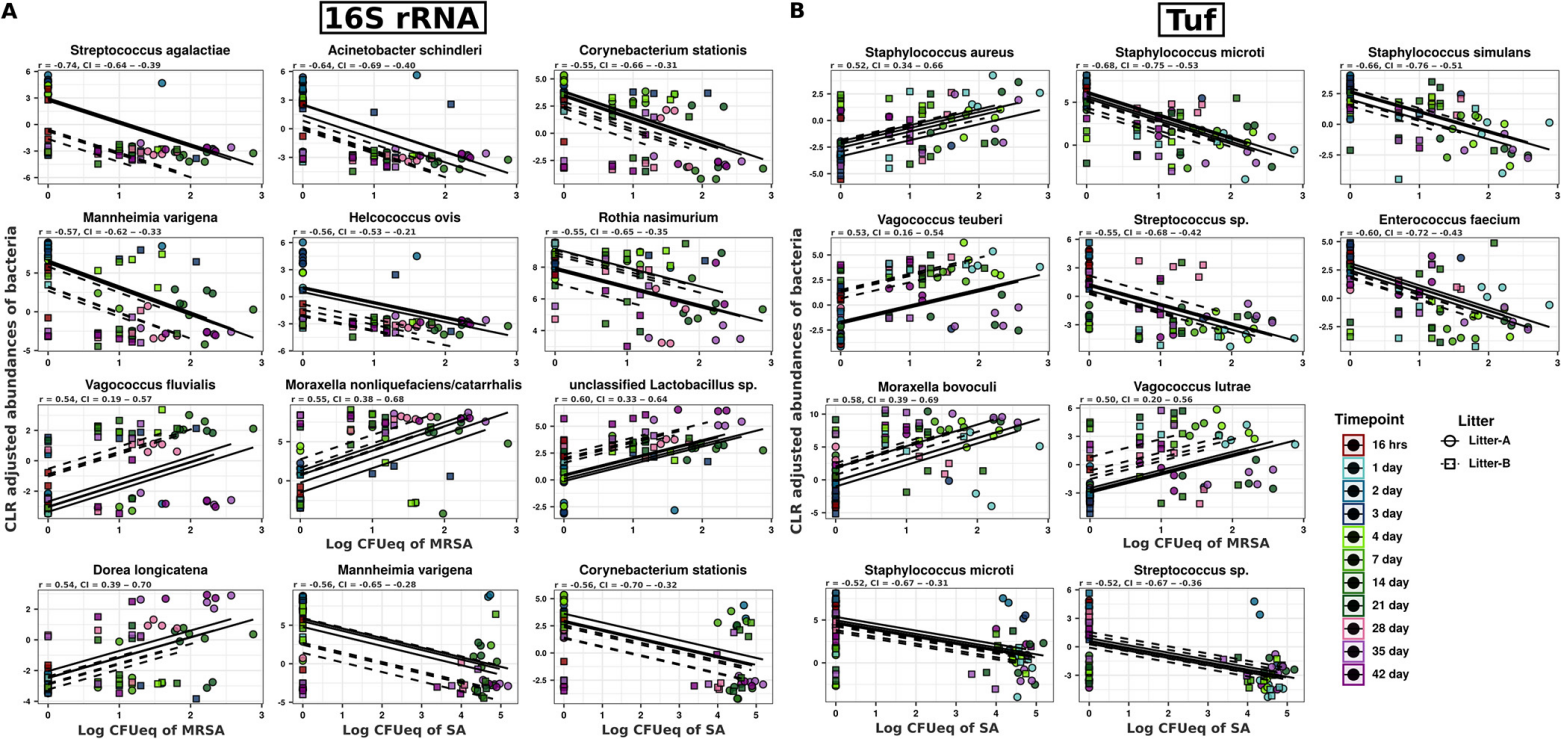
Evaluation of microbial taxa associated with nasal colonization of MRSA and S. aureus in growing piglets. (A and B) The scatterplot displays the most negatively correlated and positively correlated species-level taxa in (A) 16S rRNA and (B) *tuf* gene sequencing data. Longitudinal measurements and correlation trends are drawn per individual animal by their litter (litter A, solid line; litter B, dashed line), and correlation statistics for each species are provided above the plot (r, rmcorr correlation coefficient [r_rm_ coefficient]; CI, 95% confidence interval). Each black line corresponds to a modeled slope for each individual animal across the time point as calculated with the rmcorr package.

The 16S rRNA and *tuf* gene sequencing data provided species-level resolution for some, but not all, of the ASVs. In total, 28 different species were significantly correlated with colonization of MRSA and S. aureus (Fig. 2A; Table S1). Consistent with what we obtained at the genus level, rmcorr analysis demonstrated species such as Streptococcus agalactiae, Acinetobacter schindleri, Mannheimia varigena, Helcococcus ovis, Corynebacterium stationis, and Rothia nasimurium (r_rm_ > −0.55; adj. *P* value < 0.05) to be strongly anticorrelated with MRSA colonization in 16S data (Fig. 3A and Fig. S7). Similarly, *C. stationis* and *M. varigena* were found to be anticorrelated with S. aureus colonization (r_rm_ > 0.55; adj. *P* value < 0.05). Although a low level of *C. stationis* was also observed in MRSA-positive samples, we exclusively observed increased *C. stationis* abundance in MRSA-negative samples (Fig. S7). Of note, there was a weak but insignificant correlation of the genus *Corynebacterium* with carriage of MRSA (r_rm_ = −0.31; adj. *P* value > 0.05) and S. aureus (r_rm_ = −0.32; adj. *P* value > 0.05).

10.1128/mSystems.00152-21.7 FIG S7 Relative abundance of the taxa which displayed the most positive and negative correlation with MRSA colonization in the 16S data. Download FIG S7, PDF file, 0.6 MB. Copyright © 2021 Patel et al. 2021 Patel et al. https://creativecommons.org/licenses/by/4.0/ This content is distributed under the terms of the Creative Commons Attribution 4.0 International license.

Of the 40 species, 18 were significantly correlated with nasal colonization of MRSA and S. aureus in the *tuf* data set (Fig. 2B, Table S1). As expected, Staphylococcus aureus was positively correlated with MRSA (r_rm_ = 0.52; adj. *P* value < 0.001) and S. aureus (r_rm_ = 0.44; adj. *P* value < 0.001) nasal carriage (Fig. 3B). Apart from this, Staphylococcus hominis, Staphylococcus pettenkoferi, Staphylococcus epidermidis, and Staphylococcus cohnii also displayed significant positive correlation with carriage of MRSA (Fig. 3B and Fig. S8). In contrast, other Staphylococcus species, such as Staphylococcus microti *and* Staphylococcus simulans were found to be anticorrelated with MRSA colonization (r_rm_ > −0.50; adj. *P* value < 0.05). In addition, Moraxella bovoculi, Vagococcus teuberi, and Vagococcus lutrae were most positively correlated (r_rm_ > 0.50; adj. *P* value < 0.05), while Enterococcus faecium and Streptococcus spp. were the most negatively correlated (r_rm_ > −0.50; adj. *P* value < 0.05) with nasal colonization of MRSA (Fig. 3B and Fig. S8).

10.1128/mSystems.00152-21.8 FIG S8 Relative abundance of the taxa which displayed the most positive and negative correlation with MRSA colonization in the *tuf* data. Download FIG S8, PDF file, 0.9 MB. Copyright © 2021 Patel et al. 2021 Patel et al. https://creativecommons.org/licenses/by/4.0/ This content is distributed under the terms of the Creative Commons Attribution 4.0 International license.

To confirm the detected correlation-based associations, we performed logistic regression analysis to correlate MRSA/S. aureus colonization with the genus and species level microbiota. As expected, taxa identified as most significantly associated with MRSA/S. aureus colonization using correlation-based associations were further validated with the regression-based analysis. Genera and species found to be significantly associated with MRSA/S. aureus colonization using 16S- and *tuf*-based data sets are provided in Table S2.

10.1128/mSystems.00152-21.10 TABLE S2 Genus- and species-level taxa significantly associated with MRSA/S. aureus colonization using Maaslin2 in the 16S and *tuf* data sets. Download Table S2, XLSX file, 0.02 MB. Copyright © 2021 Patel et al. 2021 Patel et al. https://creativecommons.org/licenses/by/4.0/ This content is distributed under the terms of the Creative Commons Attribution 4.0 International license.

### Microbial taxa anticorrelated with MRSA/S. aureus nasal colonization tend to cooccur.

**FIG 4:**
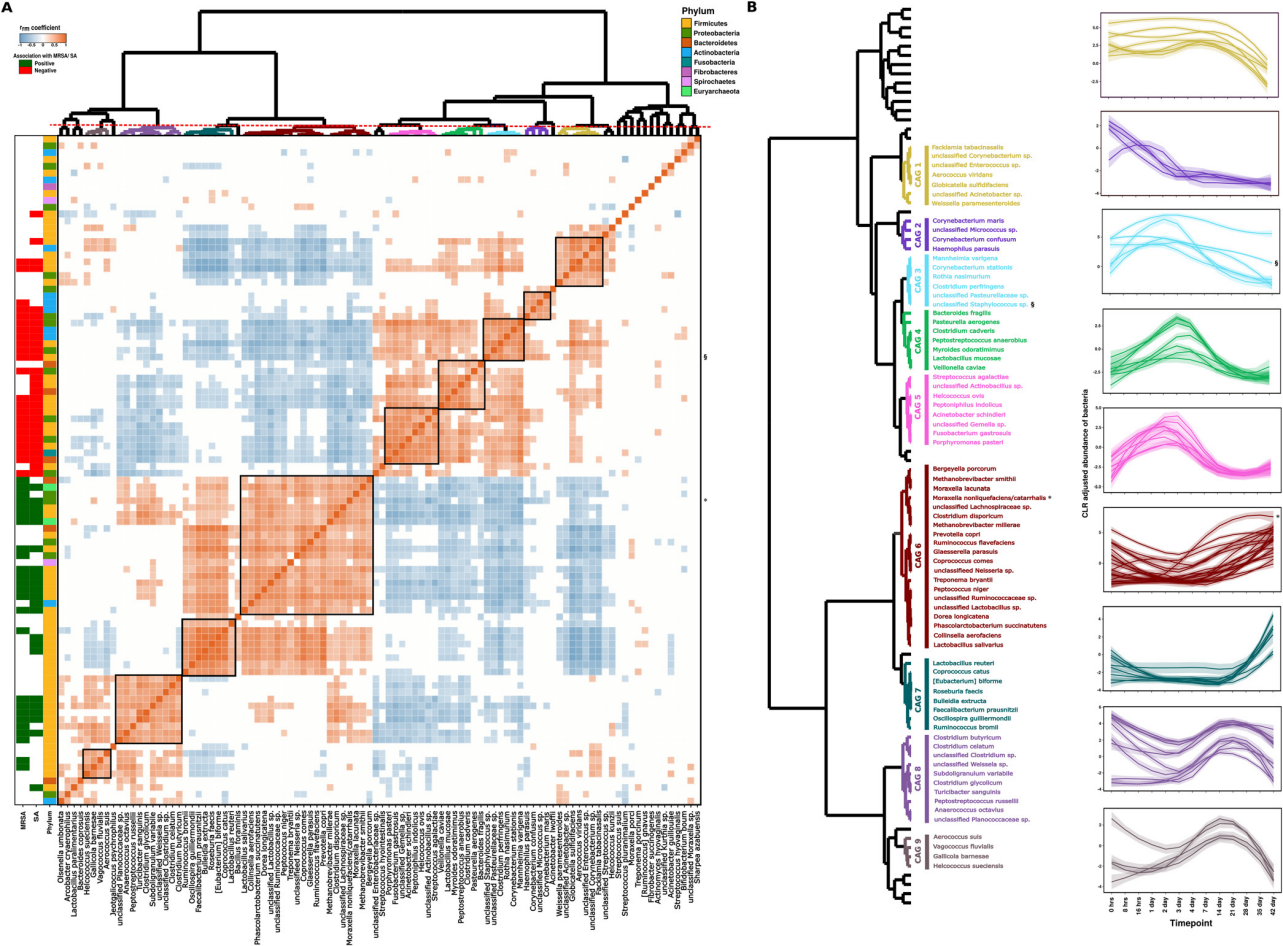
Longitudinal dynamics of bacterial species comprising coabundant groups (CAG) in 16S rRNA gene sequencing. (A) Heatmap plot of the r_rm_ coefficient values between each pair of species-level taxa. CAGs were obtained based on clustering of r_rm_ coefficient values by Spearman correlation and ward linkage hierarchical clustering. Cutting the dendrogram at a height of 1.0 allowed us to identify nine different CAGs. Taxa showing significant association with MRSA/S. aureus nasal colonization as measured by the rmcorr package are displayed as sidebars (r_rm_ coefficient). A phylum-level grouping of each individual species is displayed as the leftmost side bar. (B) Longitudinal dynamics of each species based on their identified CAGs across the time points. Species comprising different CAGs have been identified and annotated on a dendrogram based on their CAG assignment. Each individual line chart displays within-CAG dynamics of bacterial species across the time points, and the colors of the lines are matched according to their CAG assignment. Each line represents a single species.

As the nasal cavity is a nutrient-limited environment, the composition of nasal microbiota can be modulated by interactions between different bacterial species. Intermicrobial interactions can be a major driver of microbial community composition, and understanding such interactions can unveil important insights regarding establishment and carriage of MRSA/S. aureus in the nasal environment. Thus, we further investigated if microbial taxa identified as negatively associated with MRSA/S. aureus nasal colonization display a tendency toward cooccurrence or not. Using 16S species-level data, we identified nine Coabundant groups (CAGs), each comprising bacteria significantly correlated with each other from 16 h to day 42 (Fig. 4A). Of these, CAG 6, CAG 7, CAG 8, and CAG 9 were composed of taxa which were positively correlated (MRSA/S. aureus-positive CAGs), while CAG 1, CAG 2, CAG 3, CAG 4, and CAG 5 were composed of bacterial taxa which were negatively correlated with MRSA/S. aureus colonization (MRSA/S. aureus-negative CAGs). The constituent taxa of the CAGs not only cooccurred in terms of overall abundances, but also varied consistently over time. In particular, CAG 1/CAG 3/CAG 4/CAG 5 were anticorrelated with CAG 6/CAG 7/CAG 8 (Fig. 4B). We noted potential driver-passenger dynamics in CAG 6 whereby *Moraxella* spp. (marked *) is the first taxa to increase in abundance over time and is then followed by the other CAG 6 species. Using this 16S amplicon, only “unclassified Staphylococcus” in CAG 3 (marked §) was classified for this genus, but since it was negatively correlated with MRSA/S. aureus levels, we believe it is of a different species than S. aureus.

**FIG 5:**
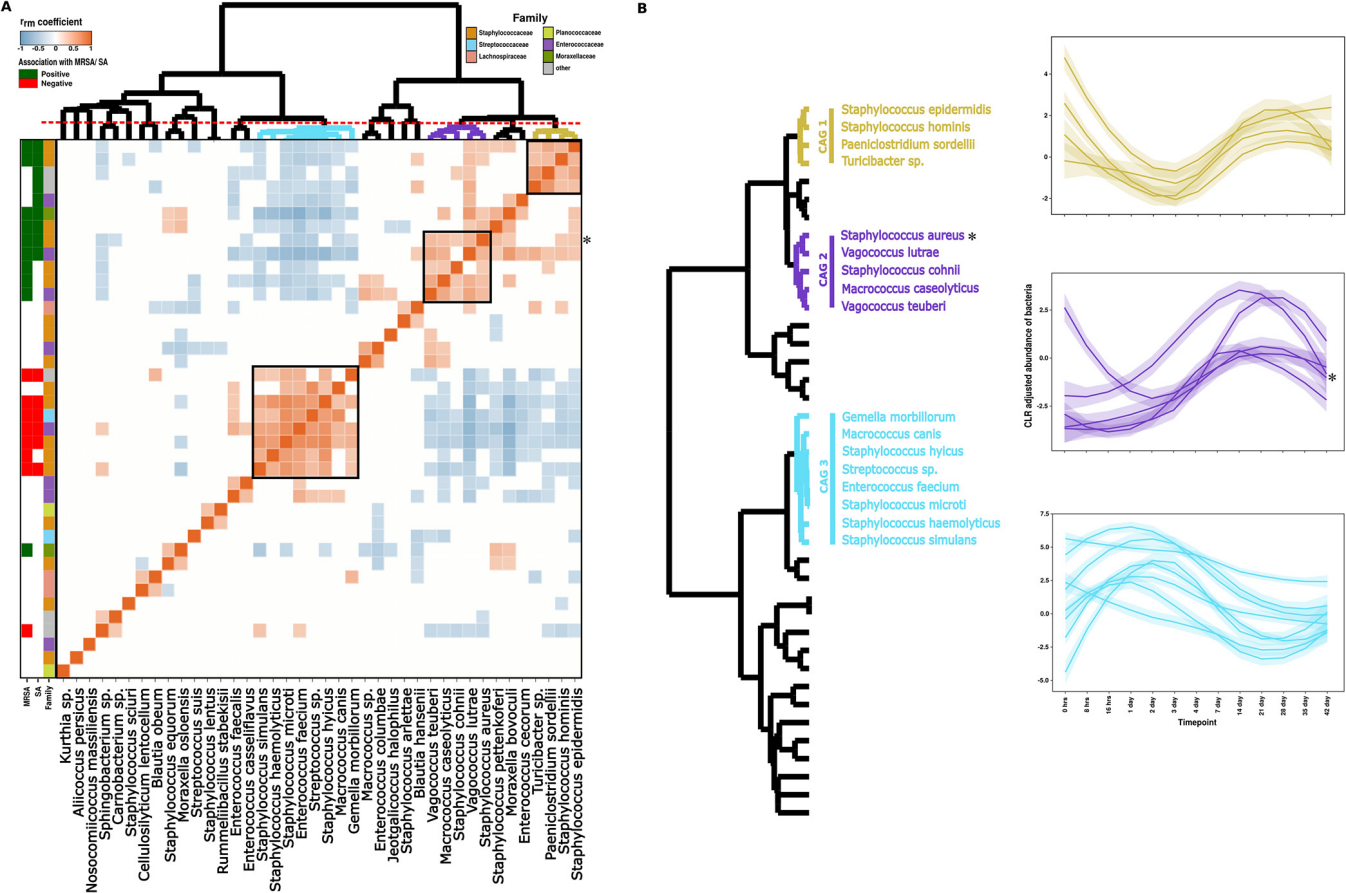
Longitudinal dynamics of bacterial species comprising coabundant groups (CAG) in *tuf* gene sequencing. (A) Heatmap plot of the r_rm_ coefficient values between each pair of species-level taxa. CAGs were obtained based on clustering of r_rm_ coefficient values by Spearman correlation and ward linkage hierarchical clustering. Cutting the dendrogram at a height of 1.0 allowed us to identify three different CAGs. Taxa showing significant association with MRSA/S. aureus nasal colonization as measured by the rmcorr package are displayed as sidebars (r_rm_ coefficient). Family-level grouping of each individual species is also displayed as the leftmost side bar. (B) Longitudinal dynamics of each species based on their identified CAGs across the time points. Species comprising different CAGs have been identified and annotated on a dendrogram based on their CAG assignment. Each individual line in the color of its CAG assignment displays the dynamics of the CAG species across the time points.

A total of three CAGs in *tuf* species-level data were identified, where CAG 1 and CAG 2 (containing S. aureus marked *) were positively correlated. CAG 3, on the other hand, was negatively correlated with MRSA/S. aureus nasal colonization (Fig. 5A). This CAG was the largest cluster containing taxa such as *S. microti*, *S. simulans*, E. faecium, and Streptococcus spp., and we observed no obvious driver-passenger dynamics in this potential MRSA-excluding group. Interestingly, Staphylococcus cohnii was correlated with S. aureus in CAG 2, while S. epidermidis and S. hominis were part of a separate CAG, suggesting differences in their abundance dynamics over the course of time (Fig. 5B).

## DISCUSSION

### Species anticorrelated to S. aureus and MRSA were identified.

Detection of S. aureus in marker gene analysis can be hampered by the fact that the piglet nostrils harbor relatively small amounts of S. aureus. The low abundance of S. aureus observed in this study is in concordance with previous findings (20, 21). The genus- and species-level resolution obtained from the sequencing data, substantiated with S. aureus-specific qPCRs and MRSA-specific culturing allowed successful identification of genera and species, which anticorrelated with S. aureus and MRSA. Interestingly, anticorrelating OTUs of *Helcococcus* and Acinetobacter have been described before in relation with low numbers of MRSA in pig noses (20), but there was no match with phyla or genera anticorrelated with MRSA in the study from Weese et al. (21). A limitation of our study is the small number of pigs that were analyzed, and it is possible that identified phyla or genera are not completely correlated with findings in other studies studying the pig microbiome.

*tuf* gene sequencing identified Staphylococcus microti and Staphylococcus simulans as negatively associated with S. aureus. Such an inhibiting effect was recently described by Brown and colleagues, showing that peptides of *S. simulans* protected against MRSA colonization and associated skin damage in a mouse model (28). These peptides were inhibiting or disrupting of the *arg*-based quorum sensing of S. aureus that has been associated with colonization and virulence factor activation. Production of *arg* quorum sensing inhibiting peptides has been detected in multiple coagulase-negative staphylococci (CoNS), including *S. simulans*, from porcine nasal swabs (29). It is considered an important mechanism for bacterial interactions evoking S. aureus competition. Other competition mechanisms involved in nasal colonization, apart from the production of small molecules, include competition for adhesions sites and nutrients, antibiosis, and inducing host defenses (30).

### *tuf* gene sequencing improves Staphylococcus species resolution.

To understand the composition of the nasal microbiome and its interactions, high taxonomic resolution at the species or even strain level is needed, as identifying anticorrelating genera to MRSA could lead to misinterpretations. For example, Yan et al. showed that two species of the genus *Corynebacterium*, the species C. accollens and C. pseudodiphtericum might act differently on S. aureus colonization in the nasal cavity (31). They identified that these species showed either inhibition or stimulation of S. aureus growth *in vitro*. Therefore, in-depth analysis of individual bacterial species to find S. aureus anticorrelative species is crucial. A limitation in our study is the reliance on the V4 region of the 16S rRNA gene, as this sequence region contains low sequence diversity and is unable to discriminate S. aureus from other Staphylococcus species in microbiome analysis (26, 27). However, several studies applying *tuf* gene sequencing have shown that this gene is discriminating of all Staphylococcus species (23, 26, 27) but can also monitor shifts in abundance of clinically important Staphylococcus species in the nasal microbiome (26, 32, 33). Using *tuf* gene sequencing, we identified 22 Staphylococcus species. This is in contrast to previous work where 12 Staphylococcus species were identified, with S. equorum as the most abundant in the porcine nose (32). In our study, *S. microti* was the most abundant staphylococcal species and was predominantly present in the first week of life. Moreover, we found that it was negatively associated with S. aureus, and its abundance decreases after day 4, when stable S. aureus colonization was established. Our species-level identification highlights the added value of complementing 16S rRNA sequencing with *tuf* gene sequencing, or multiple 16S rRNA gene regions (34), in microbiome studies, especially when Staphylococcus species are a target. To achieve even higher resolution and also functional information, metagenomic shotgun sequencing would be required.

### Trends in the developing nasal microbiome.

Here, we captured the dynamic and longitudinal development of the nasal microbiota of piglets. The identification of CAGs of bacteria also demonstrated time-dependent trends, further supporting that the porcine nasal microbiota is not stable but develops throughout time with a succession of coabundant species. The finding that the *Proteobacteria* and *Firmicutes* were the most abundant phyla agrees well with previous nasal microbiota studies of pigs (21, 32, 33, 35–37). A large drop observed in the relative abundance of *Actinobacteria* after day 7 has also been described (36). Moreover, the rise in abundance of *Proteobacteria* after weaning relates to *Moraxella* becoming the most abundant genus, and this finding is in line with the increase of *Moraxella* and *Bergeyella* upon removal of perinatal antimicrobials (35). But it is important to note that neither the piglets nor the sows received any antimicrobials for our study. Additionally, *R. nasimurium* from the phylum *Actinobacteria* has been previously described as a commensal on porcine tonsils and capable of producing the antibiotic valinomycin (38). The onset of the *R. nasimurium* decrease was around the time that S. aureus was detected and coincided with the decrease of the taxa from *tuf*-CAG 3, 16S-CAG 3, 16S-CAG 4, and 16S-CAG 5, consisting of additional anticorrelating species to S. aureus and MRSA colonization. Moreover, the taxa of 16S CAGs 6 and 7 were positively correlated with MRSA and contained the genera *Oscillospira*, *Dorea*, *Peptococcus*, *Lactobacillus*, *Coprococcus*, and *Methanobrevibacter*. This hints at a microbial shift associated with a loss of a protective effect against, or a stable colonization of, S. aureus around this time point. The question remains whether this shift is universal or an effect of host or environmental stimuli. No farm-related effects could be studied here, as the piglets were obtained from a single farm. Other environmental effects that might explain the microbial shift could be fecal input, as evident by an increase in the genus *Clostridium* around day 14, and other gut-related genera from 16S-CAG 6, 7, and 8. A decrease of maternal immunity after the first week of life, dietary changes approaching weaning, or applied perinatal antimicrobials are other factors that can modulate microbial shifts in the microbiome (39). This indicates that phyla and genera negatively associated with MRSA identified *in silico* will require further investigation with regard to their interactions with MRSA and their ecological context in the microbiome of the host.

In the human gut, the importance of an initial priming effect of natural birth on the further development of the microbiome and host immunology has been well described (40, 41). As we showed that the microbiome is shaped by development of the piglets, we expect that manipulations of the microbiota in early life could later in life stabilize in the microbiome. It is important that these manipulations will not result in dysbiosis and enable colonization of pathogenic bacteria. This underlines why longitudinal investigation of a priming effect and the developing and stabilizing community in the nasal microbiome is essential. Microbes from the maternal gut, birth canal, and skin, are the first to colonize the naive nose epithelium of the newborn piglets. Some of the species present at initial time points were found throughout the study, indicating that development of the microbiome started directly at birth and stabilized over time. Detection of *Archaea* and anaerobic bacterial species at the later time points might indicate continuous introduction of fecal species into the nostrils of piglets. This could be a result of the rooting behavior of piglets. However, recent studies have described a large archaeal diversity in the human nose (42), and *Archaea* might be a stable constituent of the porcine nasal microbiome. As the number of longitudinal pig microbiome studies from birth is extremely low, more research is needed to understand the drivers of the development of the porcine nasal microbiome. Our study observed a potential early-in-life protective delay of MRSA colonization. We identified CAGs of species negatively associated with MRSA. Members of these CAGs were present at all time points. This could indicate that these species remain colonized and could establish a lower or negative MRSA presence later in life. Therefore, it is important to investigate the species negatively associated with MRSA in a larger number and more diverse set of animals and to obtain data from pigs that present a long-term stable MRSA-negative status.

### Conclusion.

Combining 16S rRNA and *tuf* marker gene sequencing with culture and qPCR-based quantification led to the identification of bacterial species negatively associated with MRSA and S. aureus in the pig nasal microbiome. The nasal microbiome developed with a time-dependent succession of coabundance groups that may indicate early-in-life protection of S. aureus or MRSA colonization. Supplementing this study with next-generation sequencing free of amplification bias, such as shotgun metagenomics, will potentially lead to a higher taxonomic resolution and functional insights. The higher resolution is needed to study interactions at the strain level, enabling a better understanding of the complexities of the developing nasal microbiome, which could lead to novel strategies to reduce colonization of pathogens.

## MATERIALS AND METHODS

### Animal management and sampling.

The study was performed in accordance with the Dutch law on research animal welfare and was approved and registered under 2014.II.05.036 by the Animal Ethical Committee of Utrecht University, the Netherlands. The study was carried out on a conventional farm where two random sows from different pens were selected. Eight landrace piglets from two litters (litter A and litter B) were sampled at 13 different time points. Piglets received colostrum and had access to solid feed *ad libitum*. Animals received an iron injection (200 mg per animal) at the age of 1 week as a part of normal pig-farming procedure to supplement iron deficiency. Vaccinations against mycoplasma and circovirus were performed at the age of 4 weeks. All piglets were housed in two groups of intact litters until weaning at the age of 4 weeks. As part of farm management practice, piglets from litter A were separated from the sow hours before sampling at 28 days, and piglets from litter B were moved to another pen the day after sampling at weaning. After weaning, piglets from both litters were mixed with piglets from other sows and kept in larger groups. Piglets and sows enrolled in this study did not show any illness and therefore did not receive any additional treatment or antimicrobials. A nasal swab was obtained from all piglets within the minutes after birth (*t* = 0 days) using a cotton swab (Medical Wire & Equipment, Wiltshire, United Kingdom). Swabs were also obtained at 8 h, 16 h, and 24 h (*t* = 1 day) after the first sampling, after which the piglets were sampled daily (*t* = 2, 3, and 4 days) and, finally, weekly until the piglets were 6 weeks old (*t* = 7, 14, 21, 28, 35, and 42 days). Nasal swabs were suspended in 1 ml saline supplemented with 1 mM EDTA (molecular grade; Sigma-Aldrich, the Netherlands). Suspension was subsequently subsampled in 3 aliquots for (i) microbiome analysis, (ii) real-time PCR to quantify S. aureus in general (including LA-MRSA), and (iii) bacteriological culturing to enumerate MRSA.

### Quantification of S. aureus by real-time PCR.

Two hundred μl of the nasal swab suspension was used to quantify S. aureus using quantitative real-time PCR (qPCR). Briefly, phocine herpes virus (PhHV) was added to the sample as an internal amplification control (43). DNA was then extracted with the High Pure PCR template preparation kit (Roche, the Netherlands) according to the manufacturer’s instructions, and the sample was eluted in 50 μl elution buffer. Then, 5 μl of sample DNA was used in a real-time PCR that quantified S. aureus by targeting the *femA* (44) and *nuc* (45) genes using a predefined standard curve. Quantitative results of the PCR are reported as log CFU-equivalents (CFUeq).

### Enumeration of MRSA by culturing.

A 10-fold serial dilution of the nasal swab sample suspension was prepared in phosphate-buffered saline (PBS) (Gibco, the Netherlands). Next, 100 μl of each dilution (10^−1^ to 10^−4^ dilution) was plated on MRSA selective medium (Brilliance MRSA 2 agar; Oxoid, the Netherlands) and incubated at 37°C for 18 to 24 h. MRSA-suspected colonies were counted, and the number of CFU of MRSA was calculated and reported as log CFU. One MRSA-suspected colony from each sample was confirmed as LA-MRSA by targeting the ST398-specific DNA fragment C01 (46), and methicillin resistance was tested by using a *mecA* (44) PCR. In case the C01 gene-specific PCR was negative, S. aureus-specific PCRs targeting the *femA* (44) and *nuc* (45) genes were performed.

### DNA extraction and sequencing.

DNA extraction was performed using a modified version of Mag-Mini bead-beating and a magnetic bead procedure (LGC Genomics, Berlin, Germany) as described by Wyllie et al. (47). Amplicon libraries targeting the V4 region of the 16S rRNA gene were prepared using 515F (GTGCCAGCMGCCGCGGTAA) and 806R (GGACTACHVGGGTWTCTAAT) universal primers. Sequencing was performed on an Illumina MiSeq platform using v2 chemistry (2 × 250 bp) (48). Similarly, libraries amplifying the *tuf* gene, a discriminatory target for Staphylococcus species were prepared using the oligonucleotides (23) *tuf*-F (GCCAGTTGAGGACGTATTCT) and *tuf*-R (CCATTTCAGTACCTTCTGGTAA), and sequencing was performed on an Illumina MiSeq platform using v3 chemistry (2 × 300 cycle). Nontemplate DNA extraction controls were also included in the amplification and sequencing protocol to monitor potential contamination.

### Microbiota data analysis and preprocessing.

For both the 16S rRNA and *tuf* gene sequenced data sets, read quality was checked using FastQC v0.11.5 (49). Quality filtering was performed using Trim Galore v0.6.5 (50) with the following parameters: trimming low-quality ends of the reads (–quality 20), removing adapter sequences that overlaps by 7 nucleotides (–nextera, –stringency 7), discarding sequences with <80 nucleotides (–length 80), singleton reads whereby the other pair of the read is discarded excluded from downstream analysis (–paired). Quality-filtered reads were then imported into R v3.5.0 (51) for subsequent analysis with the DADA2 pipeline v1.12 (52). Amplicon sequence variants (ASVs) for 16S rRNA data (from here on, “16S” is used for “16S rRNA gene”) were inferred using following parameters: truncLen=c(200,140), maxEE=c(1), truncQ=c(2), maxN = 0, rm.phix=TRUE. While ASVs for *tuf* data (from here on, “*tuf*” is used for “*tuf* gene”) were inferred using the following parameters: truncLen=c(240,180), maxEE=c(1), truncQ=c(2), maxN = 0, rm.phix=TRUE. Briefly, the DADA2 error correction and chimera removal step was carried out on each forward and reverse read individually and then subsequently merged. At this stage, merged ASVs with at least 251 and 370 nucleotides of length for 16S and *tuf* data, respectively, were retained. The resulting nonchimeric ASVs from the 16S data were further subjected to the second stage of chimera filtering, using reference-based chimera filtering implemented in USEARCH v11 (53) with the ChimeraSlayer Gold database v2011051967.

Taxonomy was assigned to nonchimeric sequences using the naive Bayes (NB) RDP classifier natively implemented in QIIME 2 (54). For this, the classifier was trained explicitly on the region of the gene that was sequenced and used for classification with a bootstrap confidence threshold of 80%. We used the Greengenes reference database v13.8 clustered at 99% identity for classification of 16S ASVs (55). For the *tuf* data, we prepared a custom reference taxonomy database by retrieving full-length bacterium-originating *tuf* sequences from KEGG (56) (https://www.genome.jp/dbget-bin/www_bget?ko:K02358; accessed 2019) and used it for classification of *tuf* ASVs using the method described for 16S data. Additionally, for 16S amplicon data, we used SPINGO for species-level classification wherever possible (57).

Initial preprocessing of the ASV table was conducted using the decontam (58) and CoDaSeq (59) packages, whereby potential reagent contaminants were identified and removed using the frequency-based method implemented in the decontam package. Next, we filtered out ASVs based on prevalence and abundance criteria using the codaseq.filter function from the CoDaSeq package. Only ASVs present in >10% of samples with a relative abundance of >0.0001 were retained for downstream analysis, which resulted in 368 ASVs for the 16S and 204 ASVs for the *tuf* data set. Except in the case of alpha diversity, this filtered ASV count table was used for all the downstream bioinformatic analyses.

### Statistical analysis of compositional data.

All statistical analyses and graphical representations were performed in R using the packages vegan (60), CoDaSeq (59), zCompositions (61), rmcorr (62), Ggplot2 (63), Heatmaply (64), and ComplexHeatmap (65). Moreover, GraPhlAn was used for visualization of phylogenetic trees generated from species-level summarized 16S and *tuf* data sets (66). To account for the complex compositional structure of the microbiome data and to avoid the likelihood of generating spurious correlations, we first imputed the zeros in the abundance metrices using the count zero multiplicative replacement method (cmultRepl, method = “CZM”) implemented in the zCompositions package and applied a centered log-ratio transformation (CLR) using the codaSeq.clr function in the CoDaSeq package. Because the ASV table was summarized at different taxonomic levels (from phylum to species level), we used CLR transformation on each taxonomic level separately. Alpha diversity was determined using Chao1 (richness) and Shannon index (diversity), and the nonlinear association of α-diversity with time point (as numeric) was accessed by fitting the loess splines using the Ggplot2 package. The statistically significant association of time points with alpha diversity was tested using the rmcorr package. Principal-coordinate analysis (PCA) was carried out using the prcomp function in R using the Aitchison distance matrix (CLR plus Euclidean distances). Permutational multivariate analysis (PERMANOVA [67]) was performed on the Aitchison distances with 9,999 permutations to evaluate the effect of different clinical variables (i.e., time point and litter) on the nasal microbiota composition.

### Association of microbiota data with metadata.

Since the nasal piglet microbiota during first two initial time points (0 h and 8 h) was not stable and harbors bacteria that are commonly found in animal feces, in the uterus and cervix of the sow, or in soil, we considered them relatively unstable and excluded them (*n* = 15 from 16S and *n* = 16 from *tuf*) from all statistical analyses. Associations between taxa and log CFU of MRSA and log CFUeq of S. aureus were obtained using repeated measure correlation analysis from the rmcorr package (62), which determines the relationship between two continuous variables while controlling for between-individual variance. Rmcorr identifies a common regression slope and thereby estimates the association shared among all the individuals. Most popular correlation techniques, such as Pearsons correlation, assume independence of error between observations and thus cannot be used where more than one data point is obtained from individuals. Rmcorr accounts for this nonindependence among observations in repeated measurement data by removing measured variance between individuals. Similar to the Pearson correlation coefficient, the rmcorr coefficient (*r*_rm_) ranges from −1 to +1 and reports the strength of the linear association between two variables. The rmcorr method calculates the rmcorr coefficient (r_rm_), *P* value, and a 95% confidence interval of the rmcorr coefficient by bootstrapping the samples (*n* = 100). So, when there is no strong heterogeneity across subjects and parallel lines provide a good fit, the rmcorr effect size (r_rm_) will be large, with tight confidence intervals. Next, in order to confirm the rmcorr correlation findings, we performed logistic regression analysis using multivariate analysis by linear models (MaAsLin2 v1.1.1) considering litter and animal ID as random effects and MRSA/S. aureus colonization events as categorical data (68). MaAsLin2 performs boosted, additive general linear models between metadata and microbial abundance. Boosting of metadata and selection of a model was performed per taxon. Microbial abundances were CLR-transformed at each taxonomic level to account for the compositional nature of the data. Multiple testing correction was carried out with the Bonferroni method where appropriate for all statistical tests (69).

### Coabundance analysis.

Following rmcorr correlations between each pair of species, species-level summarized taxa were clustered into the coabundant groups (CAGs) based on their CLR-transformed abundances across all the samples. Correlations were considered significant below a *q* value cutoff 0.05 after Benjamini-Hochberg (BH) multiple testing correction. Hierarchical clustering was performed using the Spearman distance matrix and ward linkage clustering to identify CAGs cooccurring with each other across all time points. Next, the dendrogram was cut at a height of 1.0 to generate nine and three different CAGs for the 16S and *tuf* data sets, respectively. Taxa comprising each CAG were plotted individually to understand longitudinal dynamics of the microbiome and its association with MRSA and S. aureus colonization.

### Data availability.

Sequence data are available under NCBI BioProject accession no. PRJNA687981.
