# Supplementary figures and images for: Differential Analysis of Longitudinal Methicillin-Resistant Staphylococcus aureus Colonization in Relation to Microbial Shifts in the Nasal Microbiome of Neonatal Piglets
Source: mSystems. 2021 Jul 20;6(4):e00152-21. doi: 10.1128/mSystems.00152-21 (PMC8407314; doi:10.1128/mSystems.00152-21)

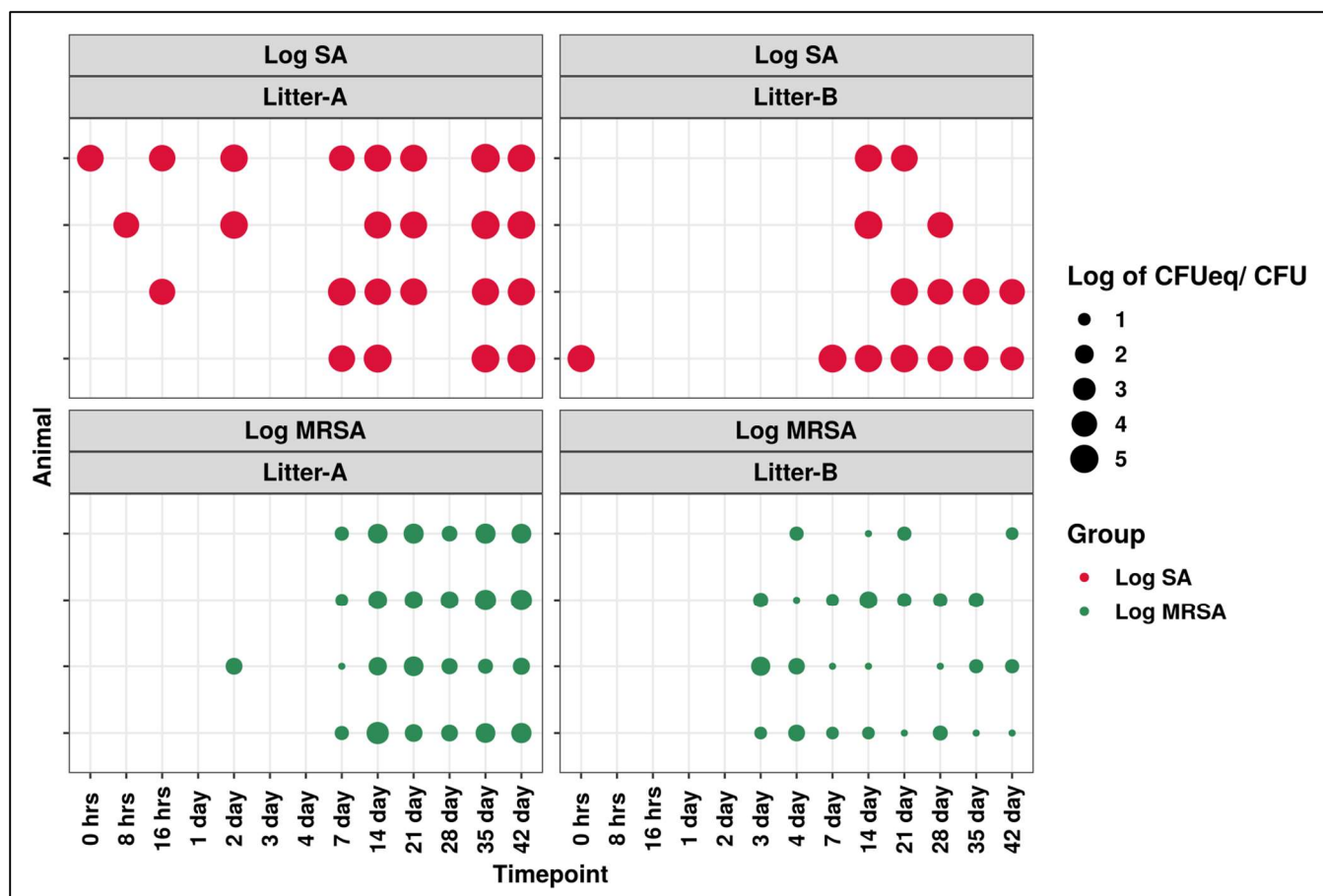

Figure S1: Summary of number of CFUeq of SA and CFU of MRSA in nasal swabs of individual piglets.

Supplement: FIG S1 [file msystems.00152-21-sf001.pdf]

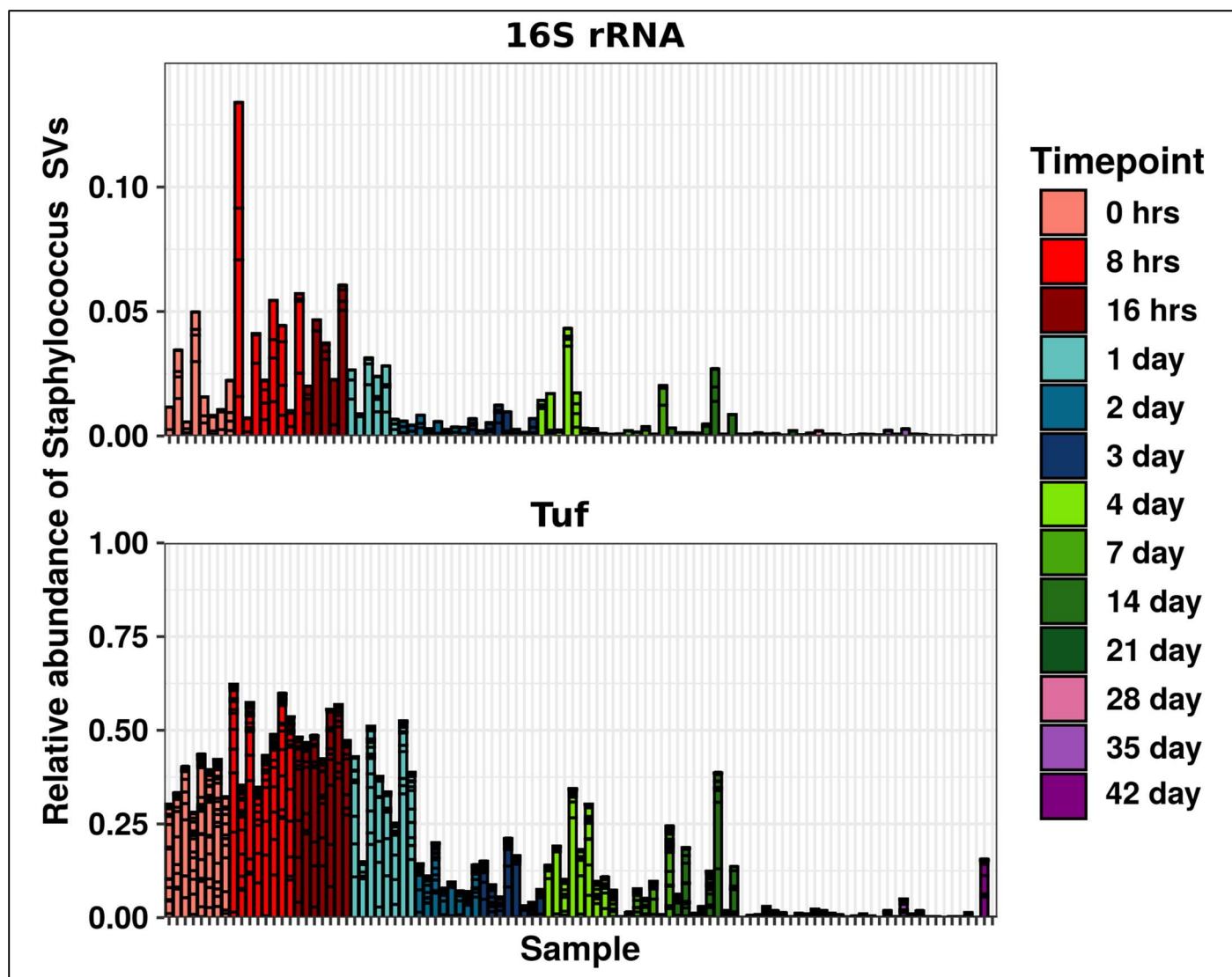

Figure S5: Relative abundance of Staphylococcus ASVs as identified from 16S and *tuf* datasets.

Supplement: FIG S5 [file msystems.00152-21-sf005.pdf]
