# Supplementary material for: Differential Analysis of Longitudinal Methicillin-Resistant Staphylococcus aureus Colonization in Relation to Microbial Shifts in the Nasal Microbiome of Neonatal Piglets
Source: mSystems. 2021 Jul 20;6(4):e00152-21. doi: 10.1128/mSystems.00152-21 (PMC8407314; doi:10.1128/mSystems.00152-21)
Supplement: FIG S2 [file msystems.00152-21-sf002.pdf]

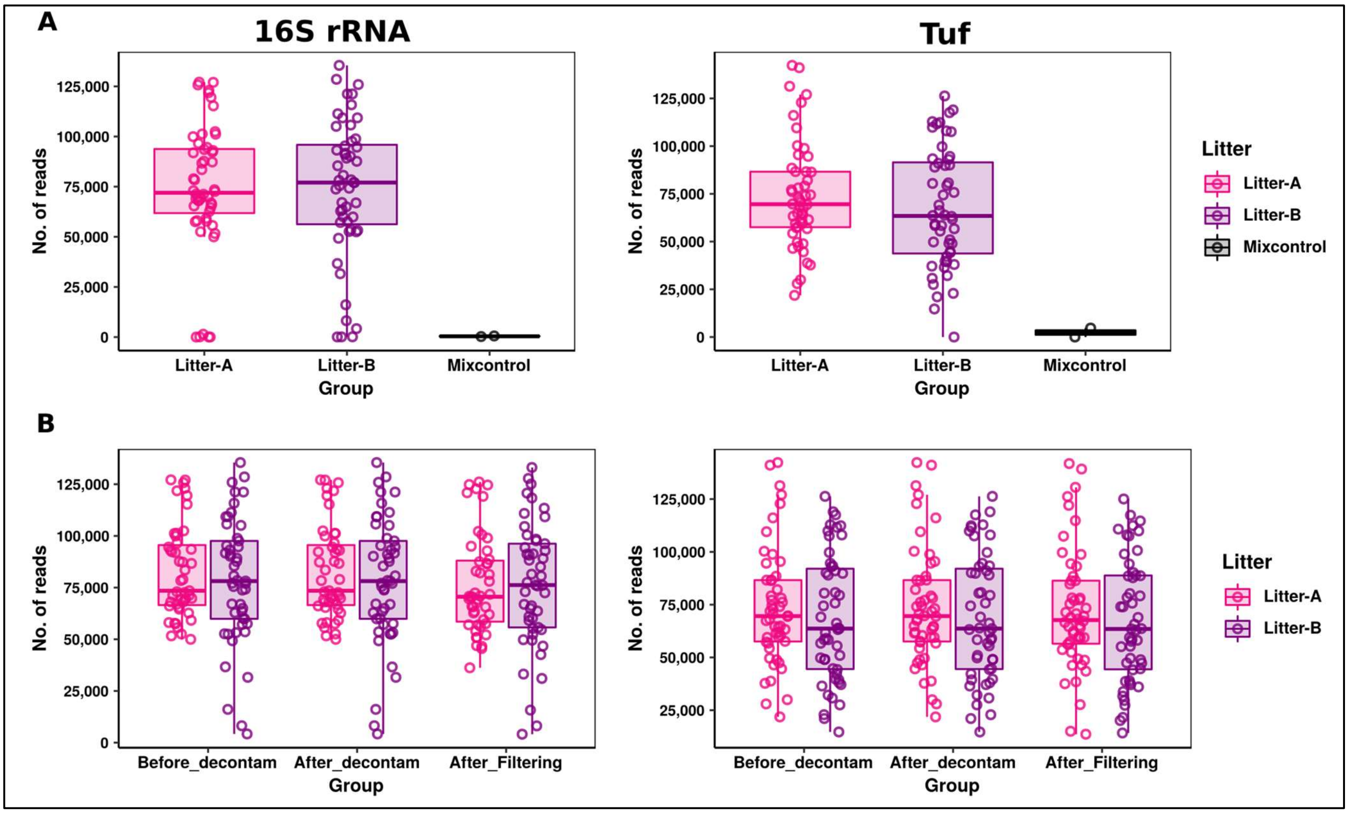

Figure S2: (A) Summary of number of sequences assigned to samples and mix-controls for 16S and *tuf* datasets. (B) Summary of sequencing depth before and after contaminant removal and after ASV filtering (i.e. prevalence and abundance-based filtering.)
