# Supplementary material for: Differential Analysis of Longitudinal Methicillin-Resistant Staphylococcus aureus Colonization in Relation to Microbial Shifts in the Nasal Microbiome of Neonatal Piglets
Source: mSystems. 2021 Jul 20;6(4):e00152-21. doi: 10.1128/mSystems.00152-21 (PMC8407314; doi:10.1128/mSystems.00152-21)
Supplement: FIG S3 [file msystems.00152-21-sf003.pdf]

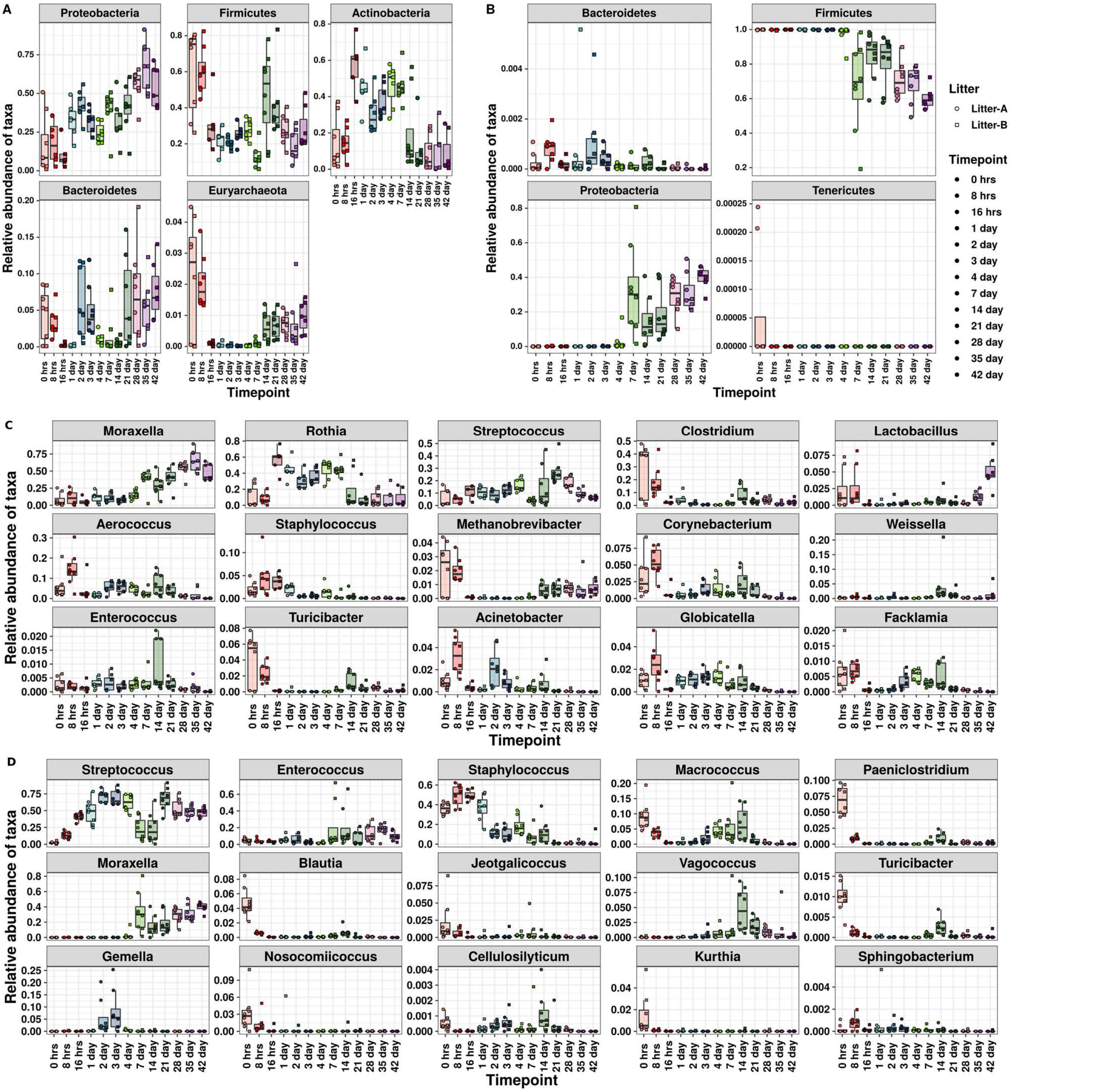

Figure S3: Distribution of topmost abundant (A) 16S phylum (B) *tuf* phylum (C) 16S genus and (D) tuf genus across the timepoints.
