# Supplementary material for: Differential Analysis of Longitudinal Methicillin-Resistant Staphylococcus aureus Colonization in Relation to Microbial Shifts in the Nasal Microbiome of Neonatal Piglets
Source: mSystems. 2021 Jul 20;6(4):e00152-21. doi: 10.1128/mSystems.00152-21 (PMC8407314; doi:10.1128/mSystems.00152-21)
Supplement: FIG S6 [file msystems.00152-21-sf006.pdf]

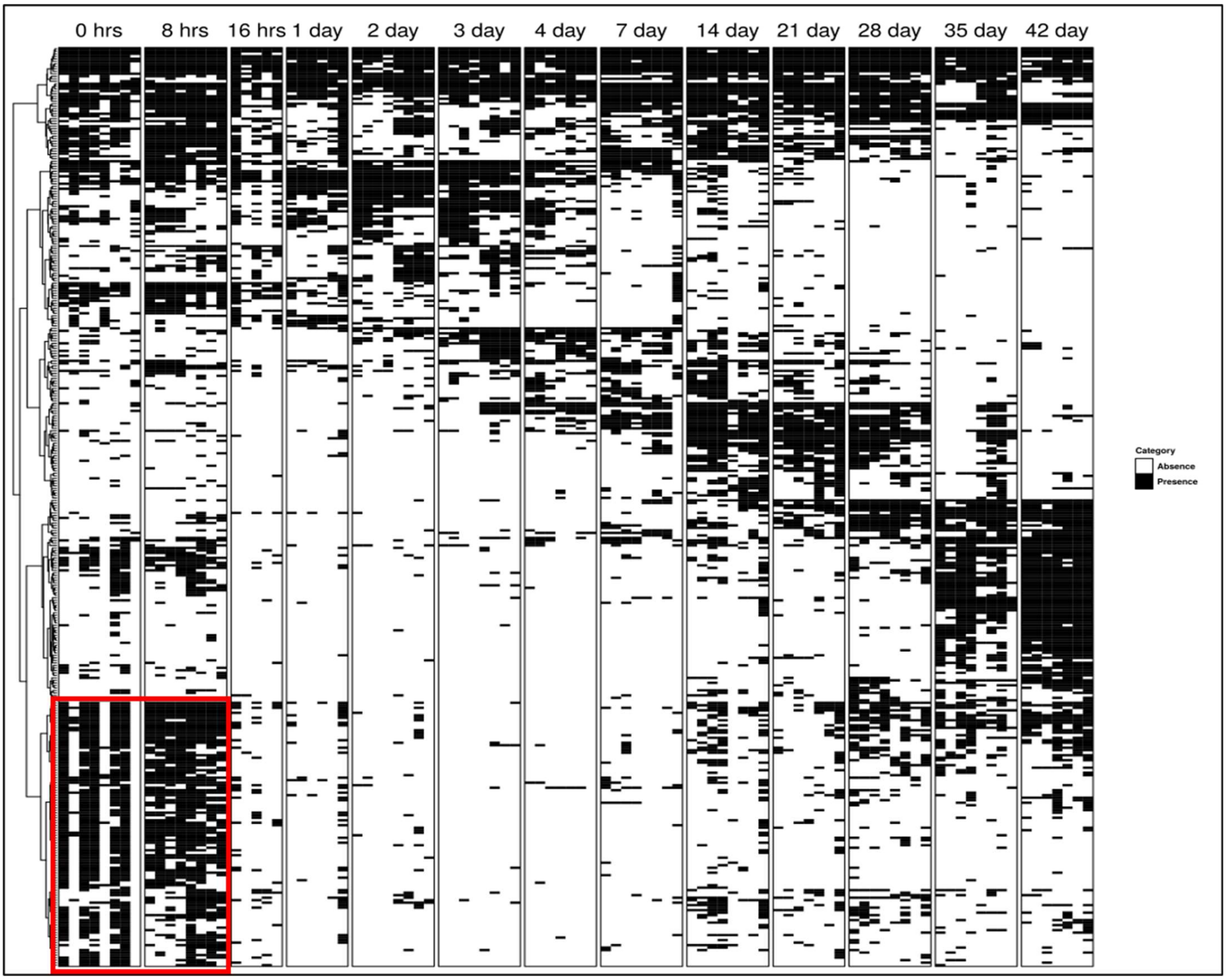

Figure S6: Presence-absence based heatmap of the top 368 ASVs in 16S data. Highlighted red block indicates ASV that are unique to the first two timepoint and belongs to the genus from *Clostridia, Lactobacillus* and *Aerococcus*.
