# Supplementary material for: Differential Analysis of Longitudinal Methicillin-Resistant Staphylococcus aureus Colonization in Relation to Microbial Shifts in the Nasal Microbiome of Neonatal Piglets
Source: mSystems. 2021 Jul 20;6(4):e00152-21. doi: 10.1128/mSystems.00152-21 (PMC8407314; doi:10.1128/mSystems.00152-21)
Supplement: FIG S8 [file msystems.00152-21-sf008.pdf]

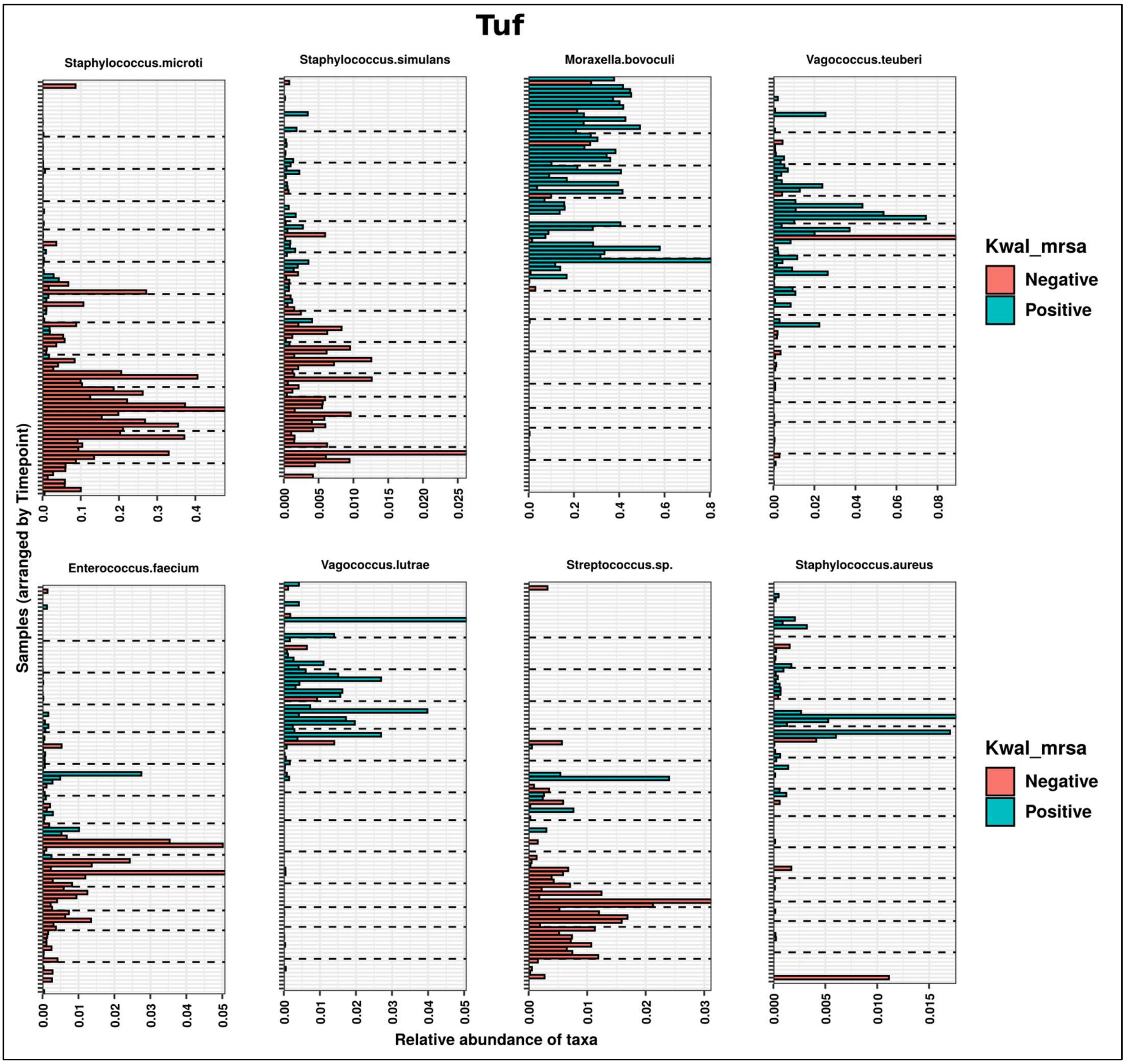

Figure S8: Relative abundance of taxa which displayed most positive and negative correlation with MRSA colonization in *tuf* data.
